# Depression, Suicide Ideation, and Thyroid Tumors Among Ukrainian Adolescents Exposed as Children to Chernobyl Radiation

**DOI:** 10.14740/jocmr2018w

**Published:** 2015-03-01

**Authors:** George Contis, Thomas P. Foley

**Affiliations:** a Medical Service Corporation International, 2000 Clarendon Boulevard, Suite 300, Arlington, VA 22201, USA; b University of Pittsburgh School of Medicine, Department of Pediatrics, Graduate School of Public Health, Department of Epidemiology, Box 38472, Pittsburgh, PA 15238, USA

**Keywords:** Adolescents, Chernobyl accident, Depression, Suicide ideation, Thyroid tumors

## Abstract

**Background:**

The Chernobyl Childhood Illness Program (CCIP) was a humanitarian assistance effort funded by the United States Congress. Its purpose was to assist the Ukrainian Government to identify and treat adolescents who developed mental and physical problems following their exposure as young children to Chernobyl radiation. Thirteen years after the Chernobyl nuclear plant accident in 1986, the CCIP examined 116,655 Ukrainian adolescents for thyroid diseases. Of these, 115,191 were also screened for depression, suicide ideation, and psychological problems. The adolescents lived in five of Ukraine’s seven most Chernobyl radiation contaminated provinces. They were up to 6 years of age or *in utero* when exposed to nuclear fallout, or were born up to 45 months after Chernobyl.

**Methods:**

Ukrainian endocrinologist and ultrasonographers used physical examination and ultrasonography of the neck to evaluate the adolescents for thyroid tumors. The adolescents were then screened for depression by the Children’s Depression Inventory (CDI). After this, Ukrainian psychologists conducted individual psychological interviews to corroborate the adolescents’ CDI responses.

**Results:**

Papillary thyroid carcinoma was diagnosed in eight adolescents, a high prevalence rate similar to that reported by other studies from the Soviet Union. Screening identified thyroid nodules in 1,967 adolescents (1.7%). Depression was diagnosed in 15,399 adolescents (13.2%), suicide ideation in 813 (5.3%), and attempted suicide in 354 (2.3%). Underlying components of the participants’ depression were negative mood, interpersonal difficulties, negative self-esteem, ineffectiveness, and anhedonia. Depression was greater in females (77%). Those with thyroid and psychological problems were referred for treatment.

**Conclusions:**

The adolescents screened by CCIP represent the largest Ukrainian cohort exposed to Chernobyl radiation as children who were evaluated for both thyroid tumors and depression. The group had an increased prevalence of thyroid cancer, thyroid tumors, depression, and suicide ideation. CCIP demonstrated that psychological problems among Chernobyl exposed adolescents began earlier in life than previously reported. They also experienced socioeconomic problems from their relocation from radiation-affected areas and from the Soviet’s inadequate responses to their health needs. CCIP’s findings underscore the requirement that governments prepare plans to deal promptly with the diagnosis and treatment of nuclear accident victims’ medical and psychological problems.

## Introduction

Following the Chernobyl nuclear disaster in 1986, the Soviet Government was slow to acknowledge the severity of the accident which killed 30 persons from acute radiation syndrome. Among those were two Russian helicopter pilots who repeatedly flew over the burning power plant dumping bags of sand in an attempt to contain the conflagration.

The Soviets did not evacuate all residents from the affected areas promptly and were slow to distribute potassium iodide prophylaxis to children exposed to high levels of iodine-131 radiation. The Government did not provide Russian citizens with timely information about the adverse effects of the disaster. As a result, many persons living in provinces affected by Chernobyl radionuclides experienced anxiety, stress, and psychological problems [1].

It was not until 4 years after the Chernobyl explosions that the Soviets finally completed the relocation of 350,000 adults and children from the most severely radiation contaminated areas [2]. While diminishing their exposure, this massive relocation of families produced significant psychosocial stress because people lost their homes, farms, jobs, and friends.

The Soviet Union’s dissolution in 1991 resulted in socioeconomic and political problems which further exacerbated the aftereffects of the Chernobyl disaster. Unemployment increased, life expectancy declined, and medical and public health services deteriorated [3]. Rumors abounded among the Russian people that those exposed to Chernobyl radiation were dying of leukemia, cancer, and suicide. Five years after the accident, the rising incidence of thyroid cancer (TCA) among radiation exposed children living in Belarus, Russia, and Ukraine was first reported to the world [4]. This gave credence to the Soviet peoples’ widespread concerns about the medical aftereffects of exposure to radiation fallout.

Subsequently, psychosocial problems including depression, post-traumatic stress disorder (PTSD), and suicide were reported among Chernobyl clean-up workers from Estonia, Ukraine, Latvia, and Kyrgyzstan [5-8]. Another study noted that married mothers of children who were evacuated from the 30 km zone around the Chernobyl plant were experiencing PTSD, depressive episodes, and high levels of distress [9].

Between 1984 - 1989 and 1989 - 1994, the adult suicide rate in the Soviet republics of Belarus, Estonia, Latvia, Lithuania, Russia, and Ukraine rose significantly [10]. A study of 4,275 Ukrainian adults, 18 years and older, found that the average onset of suicide ideation was 31 years with a lifetime prevalence of 8.2% [11].

The most well known Russian to commit suicide was Dr. Valerii Alekseevich Legasov, the Chief Deputy Director of the Kurchatov Institute of Atomic Energy. He led a team of Soviet scientists sent to Chernobyl to evaluate the causes of the explosion. Subsequently, Legasov attended the 1986 International Atomic Energy Agency (IAEA) conference on Chernobyl in Vienna. A key issue related to the Chernobyl explosions which concerned Legasov was the decision to evaluate the nuclear power rotors after the unauthorized disconnection of the plant’s safety system [12]. Two years and one day after the Chernobyl disaster, Legasov hanged himself from the stairwell of his apartment. It is believed that he committed suicide because his warnings about problems with the Chernobyl reactors had gone unheeded and his health was deteriorating from radiation exposure. Subsequently, the Soviets accepted that the design of the control rods in Chernobyl type RBMK reactors was faulty and that methodology was changed.

Reports about depression, suicide, and suicide ideation among persons exposed to Chernobyl radiation have stirred considerable scientific debate about the etiology of those problems. In 1998, a significant increase in mental retardation in addition to emotional and behavioral disorders was diagnosed in 544 Ukrainian children exposed prenatally to Chernobyl irradiation [13]. A 20-year review of perceived Chernobyl health effects concluded that there were significant psychological problems among Chernobyl survivors [14]. This report noted, however, that there was no compelling support that cognitive functioning of children was affected by exposure to Chernobyl radiation while *in utero* or at an early age. A more recent study of Chernobyl clean-up workers and evacuees found elevated levels of depression and PTSD [15]. The authors of that report postulated that the PTSD among those clean-up workers was related to “abnormal communication between the pyramidal cells of the neocortex and the hippocampus, and deep brain structures”. Unlike the well documented increased incidence of TCA among radiation exposed children, only a few studies involving small cohorts have evaluated the psychological impact of Chernobyl on that group [16].

Following the dissolution of the Soviet Government, the Ukrainian nation did not have the resources to identify and treat the emerging health problems of Chernobyl radiation exposed children. In 1998, the United States Congress mandated that foreign aid be provided to “establish ... program in Ukraine to screen and treat childhood mental and physical illness related to Chernobyl radiation”. To implement the Congressional directive, the United States Agency for International Development (USAID) funded a humanitarian assistance effort, the Chernobyl Childhood Illness Program (CCIP). This report describes the scope, activities, and findings of the CCIP.

## Materials and Methods

### Program locations and staffing

The Ukrainian Ministry of Emergencies requested the CCIP to focus its thyroid and psychological screening activities on adolescents living in five of Ukraine’s seven most Chernobyl radiation contaminated provinces (oblasts). These five provinces (Cherkassy, Kiev, Zhytomyr, Rivne, and Volyn) are in northern Ukraine along the path of the radioactive plume released by the Chernobyl explosions. The CCIP was implemented in 20 regions (raions) within these provinces: six in Cherkassy Province, three in Zhytomyr Province, six in Rivne Province, three in Volyn Province, and two in Kiev Province and city. People living in four of the five provinces (Cherkassy, Kiev, Zhytomyr, and Rivne) received approximately equal to or greater than an estimated 0.50 Grays or 50 mGy of radiation [17].

CCIP subcontracted with the ministries of health in the five participating provinces to conduct the screening and data collection activities. At one hospital in each province, CCIP established a Ukrainian American Health Center under the direction of a Ukrainian physician. The Centers served as the focal point for the participating provinces’ CCIP screening program and the base for a mobile team which performed and interpreted the psychological and thyroid examinations. Each of the five mobile teams was staffed by Ukrainian personnel consisting of three clinical psychologists, an endocrinologist, a physician/ultrasonographer, and a data entry manager/driver. The CCIP provided the Centers with laptop computers, communications equipment, a library of relevant medical publications, a van, a Hitachi portable ultrasound with a 7.5 MHz probe for thyroid imaging, a Sony image recorder, and an external power supply.

### Institutional and ethics reviews

CCIP’s institutional and ethics reviews were conducted by the Ukrainian Ministry of Emergencies and USAID. The principles outlined in the Declaration of Helsinki were followed. Parents were provided information regarding the CCIP’s objectives and gave written approval for their adolescents to be screened. Afterwards, they received a letter describing the results of the examinations and the proposed referral for treatment or follow-up of any psychological or medical problem which was identified.

### Study participants

A total of 116,655 adolescents were screened for TCA and thyroid nodules. Of these, 115,191 were also evaluated for depression by a psychological screening test and a personal interview. Of the participants, 53% were females and 47% were males.

### Thyroid screening procedures

An endocrinologist and a physician ultrasonographer conducted the thyroid gland evaluations. This involved a physical examination of the anterior compartment of the participants’ neck for a firm to hard mass or diffuse involvement, fixation on swallowing, and regional lymphadenopathy. Ultrasonography of the neck was then performed to identify abnormalities such as an isolated solid nodule or multiple nodules.

The Ukrainian endocrinologists and ultrasonographers were all experienced in the use of Sony and Hitachi ultrasound equipment. They were given additional briefings by CCIP’s US staff to ensure uniformity in the screening by ultrasonography, the interpretation of images, and the referral process for adolescents requiring follow-up care. Adolescents with suspected TCA were referred to Ukraine’s Institute of Endocrinology and Metabolism or the Center for Endocrine Surgery in Kiev for definitive diagnosis, surgery, and follow-up treatment. For individuals with a thyroid problem other than suspected TCA, referrals were made to a regional or national endocrine center for further evaluation as required by the Ukrainian Ministry of Emergencies.

### Psychological screening procedures

After the thyroid examinations were performed, the psychological screening was conducted. This consisted of the administration of the CDI and a personal psychological interview. The CDI is a test to assess self-reported symptoms of depression and its underlying components in children 7 - 17 years of age [18]. It was translated into Ukrainian from English by Ukrainian psychologists and a professional interpreter. The Ukrainian version was translated back into English by a second professional interpreter and was then validated by CCIP’s psychologists from the United States. The CDI format included five subscales which measured several components of depression: negative mood (irritability, anger), interpersonal difficulties (difficulty making/keeping close relationships), negative self-esteem (one’s belief that he/she is not good at anything), ineffectiveness (lack of motivation/inability to complete tasks), and anhedonia (loss of interest or pleasure in usual activities). The CDI listed 27 items each of which consisted of three statements. The respondent selected the one statement which best described his/her feelings for the preceding two weeks (e.g., “I feel like crying once in a while”, “I feel like crying many days”, “I feel like crying everyday”). From zero to two points were assigned to each statement, with zero representing the absence of that symptom and two representing the more advanced form of that symptom. CDI scores could range from 0 to 54, of which a score of 19 or higher generally connoted relatively severe depression [19].

The psychological screening was performed by the Ukrainian mobile teams’ clinical psychologists, all of whom had extensive education and experience in diagnosing and managing depression and stress in children. As they were not familiar with the CDI, they received instructions from CCIP’s psychologists from the United States on how to administer the test and analyze the responses. The Ukrainian psychologists were briefed on the purpose of the personal psychological interviews which were conducted after the CDI tests. They were also advised about the referral process for those adolescents with depression, suicide ideation, a history of attempted suicide, stress related issues, or other serious psychological problems.

The psychologists administered the CDI to the adolescents as a group in their classrooms or in summer camps. The tests, in pencil and paper form, were completed in 10 - 15 min and were immediately analyzed by the psychologists. The psychologists then interviewed the adolescents individually for 15 - 30 min. The purpose was to corroborate the adolescents’ CDI responses and to evaluate their psychological status in more detail, particularly if the test results indicated evidence of depression or suicide ideation.

Adolescents found to have severe psychological symptoms received immediate counseling and referral to local Ukrainian health staff for crisis intervention, psychological care, and social support. Those with moderate depression were referred to local school psychologists, regional Social Services for Youth Center psychologists and social workers, sanatorium psychologists, UNESCO Community Centers for Psychosocial Rehabilitation, and non-government organizations with psychological counseling services.

### Data procedures

At the beginning of the screening sessions, each adolescent was asked to provide basic demographic data (e.g., date of birth, place of residence) and was given an identification number. That information was entered into the mobile teams’ laptop computers by the data entry managers. If a thyroid abnormality was found by ultrasonography, the information was saved on each mobile team’s image recorder and transmitted to CCIP’s headquarters in Kiev for additional review by the Program’s US staff. The results of the CDI were retained in each provincial Center’s records and copies were sent to CCIP’s Kiev office for review by US staff.

### Dates when the CCIP was implemented

CCIP operations began in October 1999 and ended in September 2002.

### Data monitoring

Two psychologists, a public health physician, an endocrinologist, and a medical administrator from the United States designed the CCIP and visited Ukraine at least twice yearly to monitor the Program. They briefed Ukrainian and USAID personnel about the CCIP’s activities and findings, provided technical assistance to the Ukrainian implementing staff, and reviewed the Ukrainian psychologists’ and physicians’ analyses of the data.

## Results

### Thyroid screening

To clarify the relationship between age when radiation exposure occurred and the diagnosis of thyroid neoplasias by CCIP staff, the adolescents were divided into three groups based on their birthdates. Group 1 included those born up to 6 years prior to the accident (between 1980 and April 26, 1986) (57.4%). Group 2 included those who were exposed *in utero* after the first trimester of pregnancy (between April 27, 1986 and December 31, 1986) (14.1%). Group 3 included those born 9 - 45 months after the Chernobyl disaster (between January 1, 1987 and December 31, 1989) (28.5%).

Adolescents who had previously undergone thyroid surgery for thyroid cancer or were receiving care for thyroid disease did not participate in the CCIP.

CCIP screened 116,655 adolescents for thyroid tumors, of whom eight had papillary thyroid carcinoma or 1:14,582 individuals screened (Table 1). Of those with TCA, five were males and three were females. Six were born before the Chernobyl explosions and were under 6 years of age at the time of the accident (group 1). One was *in utero* when the accident occurred (group 2, born on May 2, 1986) and one was conceived about June 1, 1987 or 14 months after Chernobyl (group 3).

**Table 1:** Thyroid Cancer and Nodules Identified in Adolescents Exposed to Chernobyl Radiation in Five Ukrainian Provinces

Province	Adolescents screened (n = 116,655)	Thyroid cancer (n = 8)	Solitary nodules (n = 1,511)	Multiple nodules (n = 456)
Cherkassy	22,429	2	364 (1.6)	86 (0.4)
Kiev	4,923	0	74 (1.5)	8 (0.2)
Zhytomyr	28,521	1	310 (1.1)	120 (0.4)
Rivne	27,083	5	430 (1.6)	165 (0.6)
Volyn	33,699	0	333 (1.0)	77 (0.2)
Total	116,655	8	1,511	456

One of the children with TCA was a girl who had previously lived near the Soviet’s Beloyarsk nuclear power plant where her father was employed. When he was assigned to work at the Chernobyl nuclear power plant, she moved with him to that location. At the time the CCIP was being implemented, it was not possible to obtain information about any accidents at the Beloyarsk nuclear power plant that might have produced radiation fallout to which the girl might have been exposed.

The eight adolescents with TCA received treatment in Kiev which consisted of thyroidectomy, radioactive iodine ablation and oral thyroxine therapy. All recovered.

Of the 116,655 adolescents screened, 1,511 (1.3%) had solitary thyroid nodules and 456 (0.4%) had multiple thyroid nodules (Table 1). All were referred for medical follow-up care.

Five of the eight adolescents with TCA lived in Rivne Province which also had the largest percentage of participants with solitary nodules (430 or 1.6%) and multiple nodules (165 or 0.6%). Cherkassy Province with two cases of TCA had 364 adolescents with solitary nodules (1.6%) and 86 with multiple nodules (0.4%). Zhytomyr Province with one case of TCA had 310 adolescents with solitary nodules (1.1%) and 120 with multiple nodules (0.4%) (Table 1).

### Psychological screening

Of the 116,655 adolescents who were screened for thyroid tumors, 115,191 (98.7%) were evaluated by the CDI and by personal psychological interviews. There were 1,464 (1.3%) individuals who were examined for thyroid tumors but did not undertake the CDI or the personal interviews for nonmedical reasons (e.g., left school early).

The psychological examinations identified depression in 15,239 adolescents (13.2%) (Table 2). In addition, 813 (5.3%) had seriously considered committing suicide and 354 (2.3%) had attempted suicide. Of those with depression, 11,734 (77%) were females and 3,505 (23%) were males.

The provinces with the highest percentages of adolescents with depression were Kiev, Rivne, and Cherkassy (Table 2).

**Table 2:** Depression Identified in Adolescents Exposed to Chernobyl Radiation in Five Ukrainian Provinces

Province	Adolescents screened (n = 115,191)	Adolescents with depression (n = 15,239)
Cherkassy	23,420	2,979 (12.7)
Kiev	4,393	907 (20.6)
Zhytomyr	28,450	2,832 (10.0)
Rivne	26,573	5,022 (18.9)
Volyn	32,355	3,499 (10.8)

The psychological issues described by adolescents with depression included family members unemployed 2,956 (19.4%), financial problems 1,798 (11.8%), family member with a chronic illness 1,128 (7.4%), parents divorced 960 (6.3%), and family member recently deceased 716 (4.7%). The family composition of the adolescents with depression was no father 3,703 (24.3%), no mother 2,149 (14.1%), and no parents in the household 1,966 (12.9%).

The psychological problems described by those with suicide ideation or a history of attempted suicide included domestic violence, conflicts with non-biological parents, sibling rivalry, and alcohol abuse 718 (61.5%). Adolescents also reported experiencing interpersonal problems such as peer conflicts, relationships with the opposite sex, and low self-esteem 280 (24%). Loneliness, conflicts between expectations and capabilities, emotional deprivation, and feelings that life is not worth living were identified in 163 (14%) of those with depression. Six adolescents (0.5%) reported involvement in criminal situations.

Referrals for psychological counseling and stress management were arranged for 28,436 (24.7%) of the adolescents screened by CDI and personal psychological interviews.

### Co-morbidity

Thyroid gland and depression co-morbidity was identified in 1,828 (1.6%) of the adolescents screened. Among the psychological issues described by this group were families with financial problems 238 (13%), a family member with a chronic illness 146 (8%), and families with alcohol abuse problems 110 (6%).

## Discussion

The CCIP screened the largest cohort of Ukrainian adolescents exposed as children to Chernobyl radiation for both thyroid tumors and depression. Its findings corroborated previous reports of elevated levels of TCA and thyroid tumors among adolescents living in radionuclide contaminated areas in Belarus, Russia, and Ukraine.

The prevalence of thyroid nodules among the adolescents evaluated by CCIP was 1.7%, a rate higher than the 0.48% reported in an earlier study [20]. Thyroid nodules in children, particularly follicular adenomas, are of concern because they may become malignant [21, 22]. In addition, one study noted that thyroid nodules detected by ultrasound or the presence of diffuse goiter and elevated serum thyroglobulin are findings associated with an increased risk of TCA [23].

The CDI administered to the Ukrainian adolescents detected levels of depression, ranging between 10.0% and 20.6% among the participating adolescents. This test is specifically designed to screen for depression and components of depression among children 7 - 17 years of age. To confirm the presence of depression in adolescents, CCIP psychologist administered personal psychological interviews to the participants. Additional problems detected by CCIP’s psychologists were suicide ideation, attempted suicide, domestic violence, conflicts with non-biological parents, and alcohol abuse.

CCIP found that radiation-induced TCA in children is not the only important health aftereffect of the Chernobyl nuclear power plant accident. Depression among CCIP screened adolescents exposed to Chernobyl radiation as young children began at an earlier age than previously reported. In addition, these adolescents were also experiencing suicide ideation and attempted suicide.

During the past decade, additional medical problems have been identified among Chernobyl survivors. They include breast cancer [24], leukemia [25], bladder cancer [26], circulatory system diseases [27], diabetes mellitus [28], and genetic and birth defects among infants born to those who had been exposed to Chernobyl radionuclides [29]. These findings are in contrast to comments in reports from IAEA and other agencies which noted that TCA is the only significant medical condition resulting from exposure to Chernobyl radiation [1]. Radiation from iodine-131 and cesium-134 was no longer present after 2 years. The emerging medical problems which have been reported years after the Chernobyl accident, however, may be related to the persistence of nuclear contamination from cesium-137, strontium-90, plutonium-239, and americium-241 [1].

### Program limitations

A valid comparative population with very low estimated radiation exposure was not available to serve as a control group. Such a population could not be identified as negligible exposure to radiation did not exist in comparable socio-economic regions of Eastern Europe at the time. In addition, the CCIP was not a research study but a humanitarian assistance effort whose Congressionally mandated objective precluded the evaluation of a control group.

### Conclusions

CCIP corroborated that Ukrainian children exposed to Chernobyl radiation were at high risk for developing thyroid tumors, particularly TCA. As adolescents, they began to experience significant psychological aftereffects including depression, suicide ideation, and attempted suicide. CCIP’s findings, and recent studies on the emergence of numerous medical problems besides TCA following Chernobyl radiation exposure, are changing perceptions regard nuclear fallout aftereffects. As a result, countries with nuclear power plants must develop comprehensive plans to deal promptly with the many public health problems that will be experienced by nuclear accident victims.
